# Unapproved Pharmaceutical Ingredients Included in Dietary Supplements Associated With US Food and Drug Administration Warnings

**DOI:** 10.1001/jamanetworkopen.2018.3337

**Published:** 2018-10-12

**Authors:** Jenna Tucker, Tessa Fischer, Laurence Upjohn, David Mazzera, Madhur Kumar

**Affiliations:** 1 Food and Drug Branch, California Department of Public Health, Sacramento; 2 California Epidemiologic Investigation Service (Cal EIS) Fellowship Program, Sacramento

## Abstract

**Importance:**

Over half of adults in the United States report consuming dietary supplements. The US Food and Drug Administration (FDA) has warned of numerous dietary supplements containing undeclared, unapproved pharmaceutical ingredients. These FDA warnings have not been comprehensively analyzed for recent years.

**Objective:**

To summarize trends across adulterated (containing unapproved ingredients) dietary supplements associated with a warning released by the FDA from 2007 through 2016.

**Design, Setting, and Participants:**

In this quality improvement study, data were extracted from the FDA’s Center for Drug Evaluation and Research, Tainted Products Marketed as Dietary Supplements_CDER database from 2007 through 2016. Data from each warning were recorded unless multiple warnings were issued for the same product within a 6-month period. Date, product name, company, hidden ingredient(s), product category, source of sample, and warning document type were recorded for each included warning. Data analysis was conducted from February 2017 to June 2017.

**Results:**

From 2007 through 2016, 776 adulterated dietary supplements were identified by the FDA and 146 different dietary supplement companies were implicated. Most of these products were marketed for sexual enhancement (353 [45.5%]), weight loss (317 [40.9%]), or muscle building (92 [11.9%]), with 157 adulterated products (20.2%) containing more than 1 unapproved ingredient. The most common adulterants were sildenafil for sexual enhancement supplements (166 of 353 [47.0%]), sibutramine for weight loss supplements (269 of 317 [84.9%]), and synthetic steroids or steroid-like ingredients for muscle building supplements (82 of 92 [89.1%]). There were 28 products named in 2 or 3 warnings more than 6 months apart. Of these products, 19 (67.9%) were reported to contain new unapproved ingredients in the second or third warning, consistent with the assumption that the FDA found the product to be adulterated more than once. In recent years (2014-2016), 117 of 303 adulterated samples (38.6%) were identified through online sampling and 104 of 303 (34.3%) were identified through the examination of international mail shipments.

**Conclusions and Relevance:**

Active pharmaceuticals continue to be identified in dietary supplements, especially those marketed for sexual enhancement or weight loss, even after FDA warnings. The drug ingredients in these dietary supplements have the potential to cause serious adverse health effects owing to accidental misuse, overuse, or interaction with other medications, underlying health conditions, or other pharmaceuticals within the supplement.

## Key Points

**Question:**

What are the trends across adulterated dietary supplements associated with a warning released by the US Food and Drug Administration from 2007 through 2016?

**Findings:**

In this quality improvement study, analysis of the US Food and Drug Administration warnings from 2007 through 2016 showed that unapproved pharmaceutical ingredients were identified in 776 dietary supplements, and these products were commonly marketed for sexual enhancement, weight loss, or muscle building. The most common adulterants were sildenafil for sexual enhancement supplements, sibutramine for weight loss supplements, and synthetic steroids or steroid-like ingredients for muscle building supplements, with 157 products (20.2%) containing more than 1 unapproved ingredient.

**Meaning:**

Potentially harmful active pharmaceuticals continue to be identified in over-the-counter dietary supplements.

## Teaser

This quality improvement study examines trends across adulterated dietary supplements, containing unapproved ingredients, associated with US Food and Drug Administration (FDA) warnings.

## Introduction

In the United States, more than 50% of adults consume dietary supplements, fueling a $35 billion industry.^1,2^ Dietary supplements include vitamins, minerals, botanicals, amino acids, and enzymes that according to the US Food and Drug Administration (FDA) are not intended to treat or prevent disease.^3,4^ Under the 1994 Dietary Supplement Health and Education Act, dietary supplements were classified as a category of food and are not subject to the premarket safety and effectiveness testing required by the FDA for drugs.^5^

To identify products that are unsafe or adulterated (contain unapproved ingredients), the FDA relies on postmarket surveillance efforts including review of adverse event reports and consumer complaints, inspection of dietary supplement firms, and screening of imported products.^6^ Additionally, a dietary supplement firm is obligated to report events that require medical intervention to prevent death, hospitalization, or birth defect to the FDA.^6^ When a product has the potential to cause serious adverse health consequences, the FDA can issue a class I recall and take it off the market.^7^

One study found that dietary supplement use was associated with 23 000 emergency department visits and 2000 hospitalizations in the United States each year.^8^ Serious adverse events reported with the use of dietary supplements include stroke, acute liver injury, kidney failure, pulmonary embolisms, and death.^9^ Identifying adverse events through postmarket surveillance efforts poses some challenges, mainly owing to difficulties in asserting causality and underreporting. A US Government Accountability Office report found that, of adverse event reports received by the FDA, most do not initiate consumer protection actions like inspections or warning letters.^6^ Additionally, many consumers and physicians may not attribute symptoms to use of a dietary supplement or know to report to the FDA or the associated dietary supplement firm.^6,10^ In fact, poison control centers received over 1000 more reports of adverse events associated with dietary supplement use than the FDA did over a 3-year period.^6^

To increase transparency and public knowledge, the FDA’s Center for Drug Evaluation and Research maintains the Tainted Products Marketed as Dietary Supplements_CDER database (Tainted Supplements database) on its website as a resource to lower risk for consumers.^11,12^ This study analyzes data from the Tainted Supplements database for adulterated dietary supplements associated with a warning by the FDA from 2007 through 2016 in order to summarize trends.

## Methods

Data from 2007 through 2016 were extracted from the Tainted Supplements database on February 28, 2017. Each database entry was linked to a single FDA warning document and included the date, product name, company, hidden ingredient(s), lot, and product category (the indication for which the product was marketed). Warning document type (voluntary recall released by the responsible dietary supplement firm, public notification, news release, consumer update, or warning letter to the firm) was also recorded.

Each entry was individually reviewed. Sometimes an FDA warning document named multiple products or more than 1 warning named the same product. It is not uncommon for the FDA to release a public notification for a product that is then followed by a voluntary recall from the associated dietary supplement company or distributor, in the coming weeks to months, resulting in 2 warnings for the product in the database. In efforts to avoid double counting such products, while also capturing supplements that were repeatedly identified by the FDA over time, warnings naming the same product that were released less than 6 months apart were assumed to be action resulting from the same FDA investigation and only information from the most serious warning type released for that product was included for analysis. When warnings named the same product 6 or more months apart, they were assumed to be separate FDA investigations and were included as separate entries in the data set. This 6-month cutoff to account for company response to FDA warning is consistent with methods used in a 2014 study also focused on tainted dietary supplements identified by the FDA.^33^ For entries from 2014 to 2016, the source of the tested sample was recorded when noted in the associated warning. After the data set was cleaned, descriptive analyses were performed using SAS Enterprise Guide, version 7.1 (SAS Institute) and Microsoft Excel 2010 for Windows (Microsoft Inc).

## Results

The raw data set from the FDA’s Tainted Supplements database included 781 entries from 2007 through 2016. After review of each entry and associated warning, 14 products were added and 19 duplicates less than 6 months apart were deleted. This resulted in a total of 776 adulterated dietary supplements reported by the FDA (Table). Most adulterated products were marketed for sexual enhancement (353 [45.5%]), weight loss (317 [40.9%]), or muscle building (92 [11.9%]) (Table). There were also 14 products (1.8%) that were marketed for other indications (categorized as other), which primarily included joint or muscle pain (Table). All adulterated supplements were found to contain unapproved drug ingredients; in the majority of cases (757 of 776 [97.6%]) these ingredients were not declared on the label.

**Table.** Summary of Products Reported in the US Food and Drug Administration’s Tainted Supplements Database, 2007 Through 2016^a^

Variable	No. (%)
Total adulterated products	776 (100.0)
Year	
2007	15 (1.9)
2008	39 (5.0)
2009	173 (22.3)
2010	67 (8.6)
2011	39 (5.0)
2012	48 (6.2)
2013	92 (11.9)
2014	97 (12.5)
2015	123 (15.9)
2016	83 (10.7)
Category	
Sexual enhancement	353 (45.5)
Weight loss	317 (40.9)
Muscle building	92 (11.9)
Other	14 (1.8)
No. of hidden ingredients found	
1	619 (79.8)
2	124 (16.0)
3	25 (3.2)
4	5 (0.6)
5	1 (0.1)
6	2 (0.3)
Associated warning^b^	
Voluntary recall	360 (46.4)
Public notification	342 (44.1)
News release	58 (7.5)
Consumer update	8 (1.0)
Warning letter to firm	7 (0.9)
US Department of Justice press release	1 (0.1)

Abbreviation: CDER, Center for Drug Evaluation and Research.

^a^ Data from the US Food and Drug Administration’s Tainted Products Marketed as Dietary Supplements_CDER Database.^12^

^b^ Indicates the most serious type of warning for a given product published by the US Food and Drug Administration in a 6-month period.

The 776 entries included 746 distinct products, of which 718 products (96.2%) had a single occurrence of testing positive for adulteration. The remaining 28 products (3.8%) were tested by the FDA and found to be adulterated in 2 (26 products) or 3 (2 products) instances. Nineteen of the products (67.9%) with multiple warnings were reported to contain new ingredients in their second or third warning compared with the first warning.

**Figure 1.**
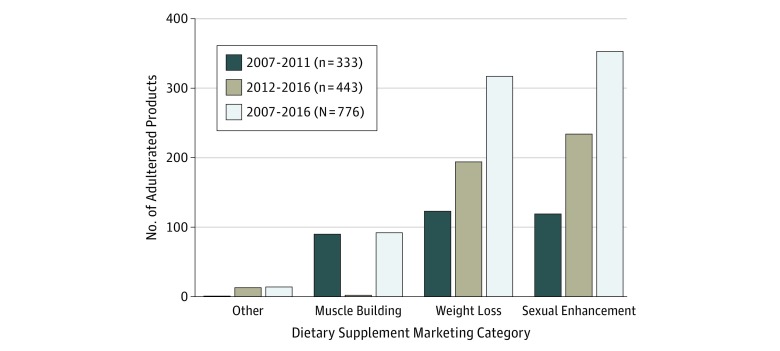
Products in the US Food and Drug Administration’s Tainted Supplements Database by Marketing Category, 2007 Through 2016 Data from the US Food and Drug Administration’s Tainted Products Marketed as Dietary Supplements_CDER (Center for Drug Evaluation and Research) database.^12^

The greatest number of products found to contain hidden ingredients were reported in 2009 (Table). This high number was primarily owing to 2 large recalls that together named 99 products. Otherwise, the highest numbers of adulterated products were identified in the most recent years of data, with 443 of 776 products (57.1%) being reported from 2012 to 2016 (Figure 1).

Most adulterated products (619 [79.8%]) were found to contain 1 unapproved drug ingredient (Table). Still, 157 of 776 products (20.2%) were found to contain more than 1 pharmaceutical, including 33 products that tested positive for 3 or more adulterants. Of these, 16 of 33 products (48.5%) were marketed for weight loss, 13 of 33 (39.4%) for sexual enhancement, and 4 of 33 (12.1%) for “other” indications. Two products, 1 marketed for sexual enhancement and 1 for joint pain, each contained 6 drug ingredients.

**Figure 2.**
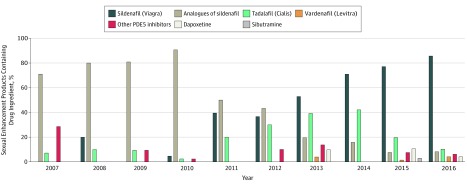
Undeclared Ingredients Identified in 353 Sexual Enhancement Products From the US Food and Drug Administration’s Tainted Supplements Database, 2007 Through 2016 Data from the US Food and Drug Administration’s Tainted Products Marketed as Dietary Supplements_CDER (Center for Drug Evaluation and Research) database.^12^ PDE5 indicates phosphodiesterase-5.

**Figure 3.**
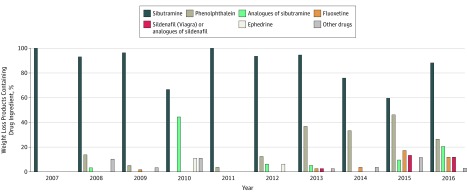
Undeclared Ingredients Identified in 317 Weight Loss Products From the US Food and Drug Administration’s Tainted Supplements Database, 2007 Through 2016 Data from the FDA’s Tainted Products Marketed as Dietary Supplements_CDER (Center for Drug Evaluation and Research) database.^12^

Certain drug ingredients were commonly detected across products marketed for the same purposes (Figure 2 and Figure 3). Overall, 287 of 353 adulterated sexual enhancement supplements (81.3%) contained sildenafil (166 of 353 [47.0%]) and/or at least 1 of its structural analogues (134 of 353 [38.0%]). Sildenafil is the active pharmaceutical ingredient in Viagra, which is a prescription medication manufactured by Pfizer Inc for erectile dysfunction.^13^ Analogues are metabolized in the body into active pharmaceutical ingredients. In the beginning of the 10-year period from 2007 through 2016, analogues of sildenafil were detected in a majority of adulterated sexual enhancement supplements (Figure 2). In 2012, however, the proportion of products containing sildenafil began to increase.

Tadalafil was also detected in 72 of 353 sexual enhancement supplements (20.4%). Tadalafil is the active ingredient in Cialis, which is manufactured by Eli Lilly and prescribed for erectile dysfunction.^14^ Vardenafil, the active ingredient in the prescription drug Levitra produced by Bayer Pharmaceuticals for erectile dysfunction, was detected in 5 products over the 10-year period.^15^ Sildenafil, tadalafil, and vardenafil are all phosphodiesterase-5 (PDE5) inhibitors and affect the same pathway in order to treat erectile dysfunction.^16^ Other PDE5 inhibitors, including PDE5 inhibitor analogues, were also detected in 27 of 353 adulterated sexual enhancement products (7.6%).

Dapoxetine, an antidepressant not approved by the FDA, was detected in 14 of 353 adulterated sexual enhancement supplements (4.0%).^17^ Two products marketed for sexual enhancement were found to contain sibutramine, a drug ingredient commonly found in weight loss supplements (Figure 3) that was removed from the US market in 2010 owing to cardiovascular risks.^18^ Overall, 65 of 353 adulterated sexual enhancement supplements (18.4%) were found to contain more than 1 hidden drug ingredient.

The most common drug ingredients detected in adulterated dietary supplements marketed for weight loss were sibutramine, sibutramine analogues, and the laxative phenolphthalein (Figure 3). Sibutramine was detected in 269 of 317 adulterated weight loss supplements (84.9%), sibutramine analogues were identified in 20 of 317 (6.3%), and phenolphthalein was identified in 75 of 317 (23.7%). Both sibutramine and phenolphthalein were removed from the US market by the FDA in 2010 and 1999, respectively.^18^ Fluoxetine, a prescription antidepressant from the same class of drugs as dapoxetine, was found in 17 of 317 weight loss products (5.4%).^19^ Sildenafil or 1 of its analogues was also identified in 12 of 317 weight loss supplements (3.8%). One adulterated weight loss product in 2010 and another in 2012 (2 of 317 [0.6%]) contained ephedrine, a stimulant that increases blood pressure and was banned from use in dietary supplements by the FDA in 2004.^20^

Sixteen of 317 adulterated weight loss supplements (5.0%) were found to contain other drug ingredients, including bumetanide, cetilistat, diclofenac, dimethylamylamine, fenfluramine, fenproporex, furosemide, lorcaserin, orlistat, phenytoin, propranolol, rimonabant, and an unspecified diuretic. In total, 80 of 317 adulterated weight loss supplements (25.2%) were found to contain more than 1 hidden drug ingredient.

A total of 92 adulterated muscle building supplements were reported. This included 73 supplements that were deemed by the FDA to contain undeclared anabolic steroids or steroid-like substances, 9 that had anabolic steroids and/or steroid-like substances declared on the label, and 10 that had aromatase inhibitors declared on the label. The aromatase inhibitors block estrogen receptors and are used in the treatment of breast cancer in postmenopausal women.^21^ Eighty-nine (96.7%) of these adulterated muscle building products reported from 2007 through 2016 were identified in just 2 years, 74 of 92 (80.4%) in 2009 and 15 of 92 (16.3%) in 2010. Overall, synthetic steroids or steroid-like ingredients were identified in 82 of 92 adulterated muscle building products (89.1%).

Fourteen adulterated supplements marketed for indications other than sexual enhancement, weight loss, or muscle building were reported by the FDA. These dietary supplements in the “other” category were marketed to assist with various conditions including joint pain, muscle pain, osteoporosis, bone cancer, sleep issues, gout, and prostate health. Half (7 of 14) of these products contained diclofenac, a prescription nonsteroidal anti-inflammatory drug, and 5 of 14 products (35.7%) contained dexamethasone, a corticosteroid commonly used to treat inflammatory conditions.^22,23^ Most (10 of 14 [71.4%]) were marketed for joint and/or muscle pain, and all but 1 of these contained diclofenac or dexamethasone. One product promoted for treating arthritis, muscle pain, osteoporosis, bone cancer, and other conditions^22^ contained both diclofenac and dexamethasone. Chlorpheniramine, an antihistamine, was detected in 3 of 14 products (21.4%). Indomethacin, a prescription nonsteroidal anti-inflammatory drug similar to diclofenac, was also detected in 3 of 14 products (21.4%). Additionally, 10 of 14 adulterated supplements in the “other” category (71.4%) contained at least 1 of 11 other drug ingredients that were identified in 1 or 2 products each: chlorpromazine, chlorzoxazone, cyproheptadine, doxepin, furosemide, phenylbutazone, ibuprofen, methocarbamol, naproxen, nefopam, and terazocin hydrochloride. More than 1 hidden drug ingredient was found in 11 of the 14 total adulterated supplements in the “other” category (78.6%).

**Figure 4.**
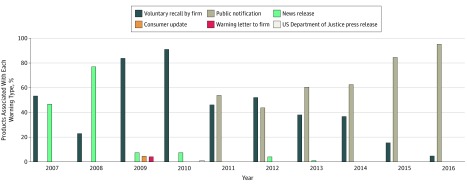
Warnings Associated With Products in the US Food and Drug Administration’s Tainted Supplements Database, 2007 Through 2016 Data from the US Food and Drug Administration’s Tainted Products Marketed as Dietary Supplements_CDER (Center for Drug Evaluation and Research) database.^12^

When assessing product warnings published by the FDA, 360 of 776 adulterated products (46.4%) were associated with a voluntary recall released by the dietary supplement firm and 342 of 776 products (44.1%) were associated with a public notification only (Table). Figure 4 shows the increasing trend of using quick, more informal warning types. In the most recent 5 years, 2012 to 2016, 321 of 443 adulterated products (72.5%) were associated with a public notification and, in both 2015 and 2016, over 80% of products were associated with public notifications as opposed to voluntary recalls.

Overall, 415 of 776 tainted products (53.5%) were associated with a specific company, with 147 different dietary supplement companies being named in total. From 2012 to 2016, a company was not identified for 306 of 443 products (69.1%). From 2014 to 2016, 117 of 303 products (38.6%) reported were identified through online sampling and 104 of 303 (34.3%) were identified through the examination of international mail shipments. The remaining 82 of 303 adulterated dietary supplements (27.1%) reported from 2014 to 2016 did not have a clear source indicated in the warning. Additionally, while this database is not intended as a mechanism for reporting adverse events linked to dietary supplement use, a few warnings did indicate that adverse events had been associated with the named dietary supplement products, including possible liver failure and death.^34,35,36^

## Discussion

The presence of pharmaceutically active ingredients in dietary supplements makes them unapproved drugs and represents an important public health concern. Of products that were found to be adulterated more than once, 19 (67.9%) had new drug ingredients reported in their second or third warning. This indicates that these products continue to be sold and are potentially dangerous even after FDA warnings. This is alarming, especially considering that the FDA is only able to test a portion of products available on the market.^12^

The most common adulterant found in sexual enhancement supplements has shifted from analogues of sildenafil to sildenafil itself in recent years. This may be because Pfizer’s patent for Viagra expired in 2012 in countries outside of the United States.^24^ It is possible that sildenafil thus became more available on the international market and was therefore increasingly used in dietary supplements marketed for sexual enhancement.

Phosphodiesterase-5 inhibitors like sildenafil have the potential to interact with nitrates found in some pharmaceutical drugs prescribed for conditions like diabetes, high blood pressure, or high cholesterol, and can lower blood pressure to dangerous levels.^25^ Men who are prescribed nitrates are contraindicated to take PDE5 inhibitors and may turn to an all-natural dietary supplement to manage erectile dysfunction, unaware that they are consuming active pharmaceutical ingredients. The prescription drugs Viagra, Cialis, and Levitra each have 1 active ingredient and come with detailed warnings, directions for use, drug-drug interactions, and contraindications. Adulterated dietary supplements, on the other hand, may contain more than 1 active pharmaceutical ingredient and lack the necessary warnings and contraindications; these products are consumed under the presumption of safety and have the potential to cause dangerous consequences in cases of misuse or overdose.

Dapoxetine, a selective serotonin reuptake inhibitor (SSRI) that has not been approved by the FDA, was also detected in adulterated sexual enhancement supplements. The use of off-label antidepressant SSRIs to treat premature ejaculation is common; however, antidepressants can increase the risk of suicidal thinking and behavior in children, adolescents, and young adults.^26,27^ The FDA data show that anyone consuming sexual enhancement dietary supplements has the potential to be unknowingly consuming PDE5 inhibitors or SSRIs, risking interaction with other medications or preexisting health conditions.

Tainted weight loss supplements were commonly adulterated with sibutramine or phenolphthalein. Sibutramine has the potential to substantially increase blood pressure or pulse rate in some patients.^28,37^ This presents a risk to patients with a history of heart disease or stroke.^28,38^ Six dietary supplements marketed for weight loss were found to contain both sibutramine and sildenafil, which lowers blood pressure. These drugs may be included in the same dietary supplement in efforts to counteract each other’s effects. Studies indicate that phenolphthalein presents a potential carcinogenic risk and may also lead to gastrointestinal disturbances or irregular heartbeat.^18,19^ It was removed from over-the-counter laxative products in the United States in 1999.^18^

Fluoxetine was also found in adulterated weight loss supplements. Fluoxetine, an SSRI antidepressant like dapoxetine, has been associated with serious adverse effects, including suicidal thinking, abnormal bleeding, and seizures.^19^ Although ephedrine was only identified in 2 weight loss supplements, it is an ingredient that the FDA banned in 2004.^20^ Even though a number of deaths were attributed to dietary supplements that contained ephedrine, it was still detected in weight loss supplements 6 and 8 years following the ban.

In 2009, 66 muscle building products were recalled in 1 release, contributing substantially to the high number of products associated with a warning that year. Following this recall, muscle building products were rarely reported as adulterated by the FDA. Most adulterated muscle building products contained anabolic steroids or steroid-like substances. Anabolic steroids have been associated with liver injury, hair loss, altered mood, kidney damage, heart attack, stroke, pulmonary embolism, and deep vein thrombosis.^29^ Some muscle building products contained aromatase inhibitors. Use of aromatase inhibitors can lead to decreased bone maturation and growth, infertility, aggressive behavior, kidney failure, and liver dysfunction.^30^ These products pose a danger to consumers, especially young people and athletes who are often the target market for muscle building products.^31^

Tainted supplements marketed for indications other than sexual enhancement, weight loss, or muscle building were most commonly adulterated with diclofenac or dexamethasone. Diclofenac is a prescription nonsteroidal anti-inflammatory drug that has the potential to increase risk of heart attack, stroke, and gastrointestinal ulceration.^22^

Overall, the number of adulterated products reported by the FDA has increased. While the FDA has focused efforts on screening of international mail shipments and online sampling in recent years, other studies have reported tainted supplements for sale at US retail locations as well.^32,33^ Adulteration with active pharmaceutical ingredients does not happen by accident and poses a serious public health risk as consumers unknowingly ingest these drugs.^9^ Adulterated dietary supplements have the potential to cause adverse health effects both on their own and also in combination with other medications an individual may be taking.

### Limitations

This analysis was performed independent of any FDA involvement. Although efforts were made to minimize editing of the original data pulled from the FDA’s website, any assumptions made about the data and/or Tainted Supplements database during the data cleaning process are those of the researchers alone.

Additionally, the total number and variety of products tested by the FDA each year is unknown. Therefore, changes in the number of products reported or the types of products reported each year could represent true changes in trends regarding adulterated dietary supplements available in the United States or they could reflect the priorities of FDA sampling, testing, and reporting efforts.

These findings are limited to the drugs for which the FDA tested. Additionally, it is possible that a few of the products subject to voluntary recall were named without having been tested by the FDA as it is not uncommon for firms to recall all of their products, in an abundance of caution, after one comes under scrutiny. Since FDA sampling has focused on dietary supplements found online or through import screenings, this data analysis does not indicate the prevalence of these adulterated supplements at retail locations.

Finally, the FDA maintains other databases on its website. Review of these pages indicates that the Tainted Supplements database contains the most consistent and complete data regarding adulterated dietary supplements, however, it is possible that warnings were posted on other FDA sites and missed in the Tainted Supplements database.

## Conclusions

Dietary supplements are not subject to premarket approval for safety and effectiveness by the FDA and some have been found to contain undeclared drug ingredients. Of products found to be adulterated more than once, the majority were reported to contain new drug ingredients in subsequent warnings, indicating that adulterated dietary supplements continue to be an issue even after FDA action.

The active pharmaceutical ingredients identified in dietary supplements are present at unknown concentrations and have not been characterized as safe and effective by the FDA, making them unapproved drugs. These products have the potential to cause severe adverse health effects owing to accidental misuse, overuse, or interaction with other medications, underlying health conditions, or other drugs within the same dietary supplement. As the dietary supplement industry continues to grow in the United States, it is essential to further address this significant public health issue.
