# Error in the Text, Figure, and Affiliations

**DOI:** 10.1001/jamanetworkopen.2018.5765

**Published:** 2018-11-30

In the Original Investigation titled “Unapproved Pharmaceutical Ingredients Included in Dietary Supplements Associated With US Food and Drug Administration Warnings,”^1^ published October 12, 2018, there were errors in the text, Figure 4, and the Author Affiliations. On page 6, in the first paragraph “phytoin” was replaced with “phenytoin” and in the third paragraph “terazin” was replaced with “terazocin.” In Figure 4, the first entry in the key should have read, “Voluntary recall by firm.” The Author Affiliations for Jenna Tucker, MPH, should have appeared as follows: Food and Drug Branch, California Department of Public Health, Sacramento; California Epidemiologic Investigation Service (Cal EIS) Fellowship Program, Sacramento. This article has been corrected.^1^

## References

[1] Tucker J, Fischer T, Upjohn L, Mazzera D, Kumar M Unapproved pharmaceutical ingredients included in dietary supplements associated with US Food and Drug Administration warnings. JAMA Netw Open. 2018;1(6):. doi:10.1001/jamanetworkopen.2018.3337 PMC6324457 30646238

